# Simple Rapid Near-Patient Diagnostics for Tuberculosis Remain Elusive—Is a “Treat-to-Test” Strategy More Realistic?

**DOI:** 10.1371/journal.ppat.1002207

**Published:** 2011-11-03

**Authors:** Alice L. den Hertog, Oleg A. Mayboroda, Paul R. Klatser, Richard M. Anthony

**Affiliations:** 1 KIT Biomedical Research, Royal Tropical Institute, Amsterdam, The Netherlands; 2 Biomolecular Mass Spectrometry Unit, Leiden University Medical Centre (LUMC), Leiden, The Netherlands; The Fox Chase Cancer Center, United States of America

An accurate, simple, and direct test for tuberculosis (TB) has been a priority for many years, but to date no such test has become available. Easily detectable sensitive and specific biomarkers are elusive and may remain so. In parallel to the essential extensive efforts in biomarker discovery performed in this field, we suggest it is also worthwhile to evaluate the utility of biomarkers of TB infection in a “treat-to-test” strategy. Biomarkers of TB infection already identified or detected in ongoing systematic studies that are not useful for simple near-patient tests may nonetheless be suitable in a treat-to-test strategy. Thus, we call for the investigation of the kinetics of these biomarkers during the early phase of treatment.

## Background

To date no simple, rapid, accurate test for TB has been developed. An effective immunologic lateral flow assay able to test blood, urine, or conceivably sputum would be a major step forwards. However, assays based on antibody detection, although highly effective for other diseases, have a dismal record for TB diagnostics in spite of decades of dedicated research. In recent years, a selection of the then available antibody-based assays were tested and shown to have no value for the diagnosis of active TB [1]. The situation with antigen-based assays may be more promising, but even with these assays progress has been limited. Antigens with acceptable specificity have been identified and tests are available to detect them, but the sensitivity is generally low. Notably, the detection of mycobacterially derived lipoarabinomannan (LAM) in urine has been shown to be specific, but, except possibly in specific groups of patients (HIV-infected with low CD4 counts), the sensitivity is currently far too low to replace microscopy [2]. As recently reviewed by McNerney and Daley (2011) [3], there is not only a need for new biomarkers, but also new detection technologies.

Because of these obstacles, molecular methods based on DNA amplification have been increasingly developed and applied. This work has resulted in a number of highly effective and innovative assays with excellent performance [4]. Unfortunately, DNA amplification technologies are fundamentally more complex than, for example, a lateral flow-based immunological assay. Thus, providing and maintaining molecular testing where it is most needed will be a huge logistical and financial challenge.

For this reason, there remains considerable effort invested in identifying suitable human and mycobacterial biomarkers for use in a simple and rapid immunological assay. Indeed, state of the art detection and bioinformatics techniques are now being applied systematically for this purpose. Recently, Berry et al. (2010) [5] published a detailed transcriptional analysis of the human response to mycobacterial infection and identified transcriptional signatures that appear to be associated with TB infection and different stages of infection. An analysis of the dominant proteins present during different phases in a *Mycobacterium tuberculosis* infection model in guinea pigs was recently published by Kruh et al. (2010) [6], and proteomic analysis revealed that highly immunogenic TB antigens were released in exosomes of TB-infected macrophages [7]. Kunnath-Velayudhan et al. (2010) [8] studied the antibody response of patients at different stages of the disease and documented marked antibody target preferences between patients, as well as a correlation of the response with disease burden.

These efforts to characterize antibodies and other indicators of TB disease are essential, but the failure to date to identify a diagnostic biomarker, admittedly with less sophisticated tools, suggests to us that discovery of suitable biomarkers and the development of a useful test for near-patient diagnostic use in the immediate future is far from certain. That is why we would like to take this opportunity to call for the consideration of the investigation of a parallel pragmatic strategy that may allow the development of a clinically useful assay in a shorter period.

## Discussion

We would like to suggest that the use of immunological assays be considered in a treat-to-test strategy. In this approach, TB suspects would be started on treatment empirically, and after a number of days, a test would be performed to measure mycobacterial or host biomarkers.

There is evidence available supporting this proposition. A proportion of patients beginning TB treatment suffer a so-called paradoxical response, which is presumably an immunological reaction to the burst of mycobacterial antigens released when treatment starts [9], [10].

Mattos et al. (2010) [11] recently investigated the levels of specific antibodies in patients with active TB or after 3 or 6 months of treatment. They identified an increase in serum levels of antibodies against an intracellular antigen (the 16-KDa alpha crystallin) during therapy in many patients. This antibody response presumably followed the release of intracellular mycobacterial antigens as a result of the initial wave of bacterial killing. Indeed, it has been established that upon initiation of appropriate TB therapy, the majority of the mycobacteria present are killed in the first few days of therapy [12].

**Figure 1:**
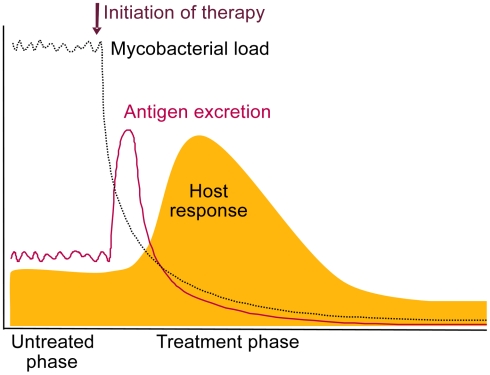
Schematic representation of the expected release of TB antigens and consequential host antibody response upon initiation of treatment. We propose that measurement of these increased levels can be useful for diagnosing TB in a treat-to-test strategy.

Measurement of antigens during the peak of bacterial killing (Figure 1), and thus antigen release, would in principle reduce the analytical sensitivity required for direct testing at presentation.

We also expect antibody titers to rapidly increase upon release of TB antigens, as the immune system will have been primed during initial infection (Figure 1). A relative increase in anti-TB antibody levels occurring after a short treatment period would also be indicative of true infection and effective therapy. The shift towards antibodies against intracellular TB antigens shown by Mattos et al. (2010) [11] during successful therapy strongly supports this. The timing of this response is yet to be determined and would be most interesting diagnostically if changes are detectable within 1 or 2 weeks. As antibody profiles of TB patients have been shown to vary considerably between patients [8], antibody testing may not be straightforward and may require multiplex testing.

Currently, due to the lack of better diagnostic tools, TB suspects are often empirically prescribed a short course of broad spectrum antibiotics in an attempt to rule out TB. A presumptive diagnosis of TB is made only if the symptoms remain. This “rule-out” strategy causes further delay for TB patients requiring anti-TB treatment. Also, due to the frequent inappropriate prescription of fluoroquinolones as broad spectrum antibiotics, there is a serious danger of increased resistance to fluoroquinolones, which are valuable second-line TB drugs [13]. Increased fluoroquinolone resistance has already been detected in India [14] and other countries. Our suggested “rule-in” strategy would avoid treatment delay and avoid possible mono-therapy with potential second-line drugs but would result in a number of non-TB patients receiving a few doses of multidrug TB treatment for testing.

In many locations, a significant proportion of patients are started on a full course of TB therapy on the basis of clinical suspicion without bacteriological confirmation. This occurs both in high-income and low-income settings [15]–[18], and only in some cases after a long period is therapy reevaluated.

We are aware that our suggested treat-to-test strategy is not the ideal “rapid” test, and, for example, would likely fail in patients with multidrug-resistant (MDR) or monoresistant TB strains, as these may not be differentiated from non-TB patients. Additionally, in some situations patients may be infected with multiple genotypes [19], one of which is MDR, and in this case initial killing of the sensitive isolate may result in a positive signal for this type of test. However, it should be noted that this type of mixed infection is also not easily identified using molecular methods. It was recently reported that the Xpert MTB/RIF assay requires between 65% and 100% of the DNA present to be derived from a resistant isolate to detect rifampicin resistance [20].

Nonetheless, a test that could detect treatment response, if applied in a different way, is in fact highly desirable, as patients known to be infected with TB but receiving ineffective therapy could be identified. It could also be envisioned that the extent of killing is reflected in the biomarker response, and susceptibility to only one or two drugs in the cocktail results in an intermediate response. This would ultimately mean that a biomarker response could be used to classify patients as either having susceptible TB, (M)DR-TB, or no TB. Thus, a negative result or weak response in a treat-to-test strategy could also be used to initiate further investigation for other diseases as well as testing for MDR-TB.

As a treat-to-test strategy would involve an additional visit to a diagnostic centre, as opposed to one-stop microscopy [21] or a true rapid near-patient test, there remains a risk of dropout during the diagnostic procedure. This is also true for culture and microscopy performed on multiple samples. And it should be noted that the use of broad spectrum antibiotics—particularly as agents are often inappropriately selected with activity against *M. tuberculosis*—has been shown to result in a delay in starting multidrug treatment [22], as well as inevitably some loss to follow up.

In summary, although a true direct test for TB remains a priority, biomarkers identified in ongoing systematic studies may not in the short term lead to assays with the required sensitivity and specificity. Nonetheless, we believe some of these biomarkers may be suitable in a treat-to-test strategy, so we encourage measurement of their kinetics during the early phase of treatment.
